# Supplementary material for: Medicinal Phytocompounds as Potential Inhibitors of p300-HIF1α Interaction: A Structure-Based Screening and Molecular Dynamics Simulation Study
Source: Pharmaceuticals (Basel). 2025 Apr 21;18(4):602. doi: 10.3390/ph18040602 (PMC12030413; doi:10.3390/ph18040602)
Supplement: Supplementary file 1 [file pharmaceuticals-18-00602-s001.zip › pharmaceuticals-3537506-supplementary.pdf]

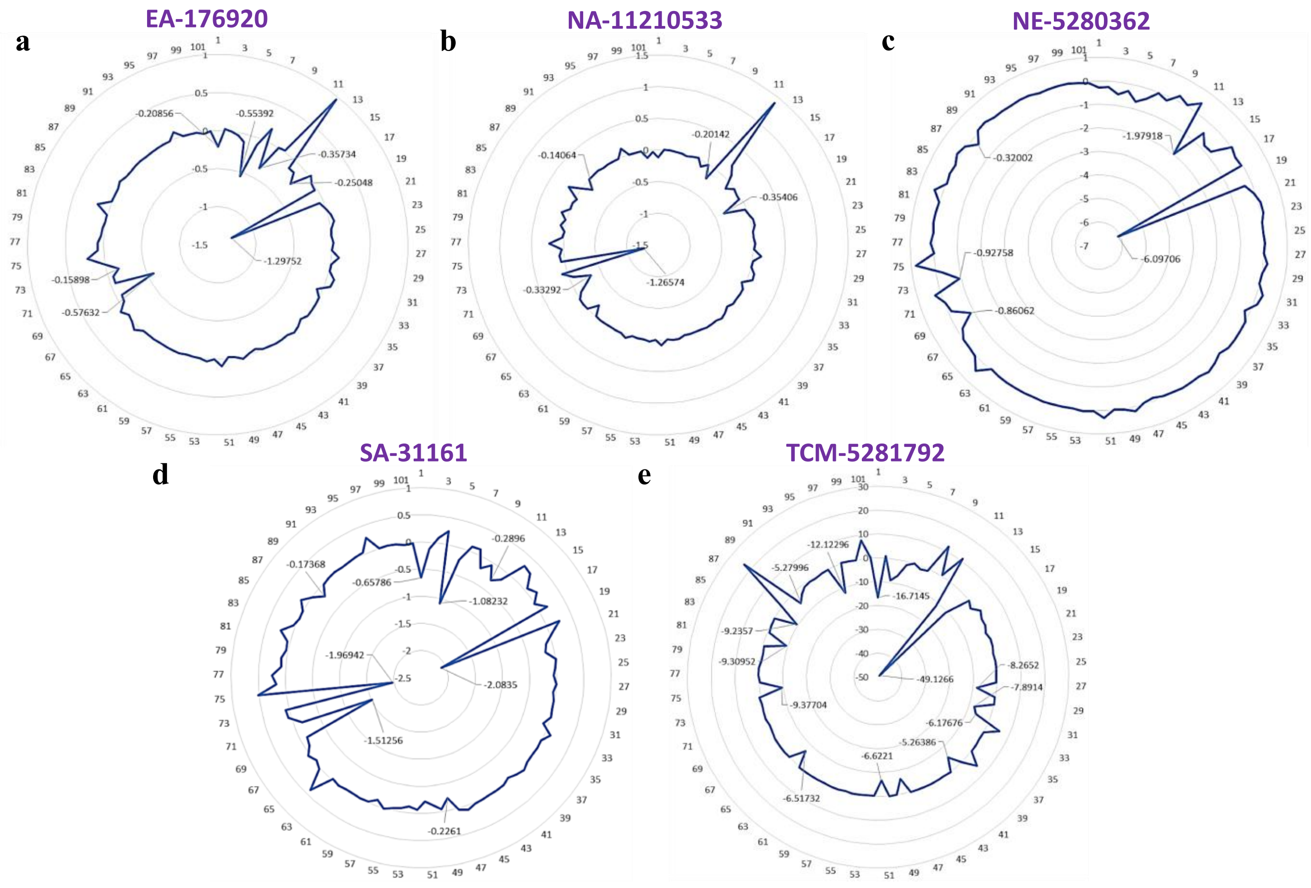

Supplementary figure S1. Binding energy of residues based on MM/GBSA decomposition. (**a**) Represents the binding energy of EA-176920-p300 complex residues. (**b**) Represents the binding energy of NA-11210533-p300 complex residues. (**c**) Represents the binding energy of NE-5280362-p300 complex residues. (**d**) Represents the binding energy of SA-31161-p300 complex residues. (**e**) Represents the binding energy of TCM-5281792-p300 complex residues.
